# A scoping review to identify process and outcome measures used in acceptance and commitment therapy research, with adults with acquired neurological conditions

**DOI:** 10.1177/02692155221144554

**Published:** 2022-12-20

**Authors:** Hannah Foote, Audrey Bowen, Sarah Cotterill, Geoff Hill, Matilde Pieri, Emma Patchwood

**Affiliations:** 1 Geoffrey Jefferson Brain Research Centre, The Manchester Academic Health Science Centre, 5292 Northern Care Alliance and University of Manchester, Manchester, UK; 2 Division of Neuroscience and Experimental Psychology, Faculty of Biology, Medicine and Health, 5292 University of Manchester, Manchester, UK; 3 Centre for Biostatistics, 5292 University of Manchester, Manchester, UK; 4 5460 South Tees Hospitals NHS Foundation Trust, The James Cook University Hospital, Middlesbrough, UK; 5 3525 Glasgow Caledonian University, Glasgow, UK

**Keywords:** Neurological conditions, scoping review, processes of change, outcomes, Acceptance and Commitment Therapy (ACT)

## Abstract

**Background:**

Acceptance and Commitment Therapy interventions are increasing in use in neurological populations. There is a lack of information on the measures available.

**Purpose:**

To identify and classify the measures used in Acceptance and Commitment Therapy research studies with adults with acquired neurological conditions.

**Methods:**

PRISMA-ScR guided scoping review. MEDLINE, PsycInfo and CINAHL databases searched (up to date 29/06/2022) with forward and backward searching. All study types included. Extraction of Acceptance and Commitment Therapy process-of-change and health-related outcome measures. Outcomes coded using the Core Outcome Measures in Effectiveness Trials (COMET) taxonomy.

**Results:**

Three hundred and thirty three papers found on searching. Fifty four studies included and 136 measurement tools extracted. Conditions included multiple sclerosis, traumatic brain injury and stroke. Thirty-eight studies measured processes of change, with 32 measures extracted. The process measure most often used was the Acceptance and Action Questionnaire (*n* = 21 studies). One hundred and four health-related outcome measures extracted. Measures exploring quality of life, health status, anxiety and depression occurred most frequently, and were used in all included neurological conditions. COMET domains most frequently coded were emotional functioning/well-being (*n* = 50), physical functioning (*n* = 32), role functioning (*n* = 22) and psychiatric (*n* = 22).

**Conclusions:**

This study provides a resource to support future identification of candidate measures. This could aid development of a Core Outcome Set to support both research and clinical practice. Further research to identify the most appropriate and relevant targets and tools for use in these populations should include expert consensus, patient, carer and public involvement and psychometric examination of measures.

## Introduction

Mental health needs are commonly unmet in people with neurological conditions,^1,3^ and developing interventions to support wellbeing is a global research priority.^4,8^ Acceptance and Commitment Therapy is a trans-diagnostic approach^4^ that shows promise.^5,7^ There is evidence, for example, trials in multiple sclerosis (MS)^8,11^ and traumatic brain injury (TBI),^12,13^ of decreased anxiety^11^ and psychological distress,^12,13^ and increased acceptance.^9,10^ However, it is unclear which measures are available for process of change and outcomes.

**Figure 1.**
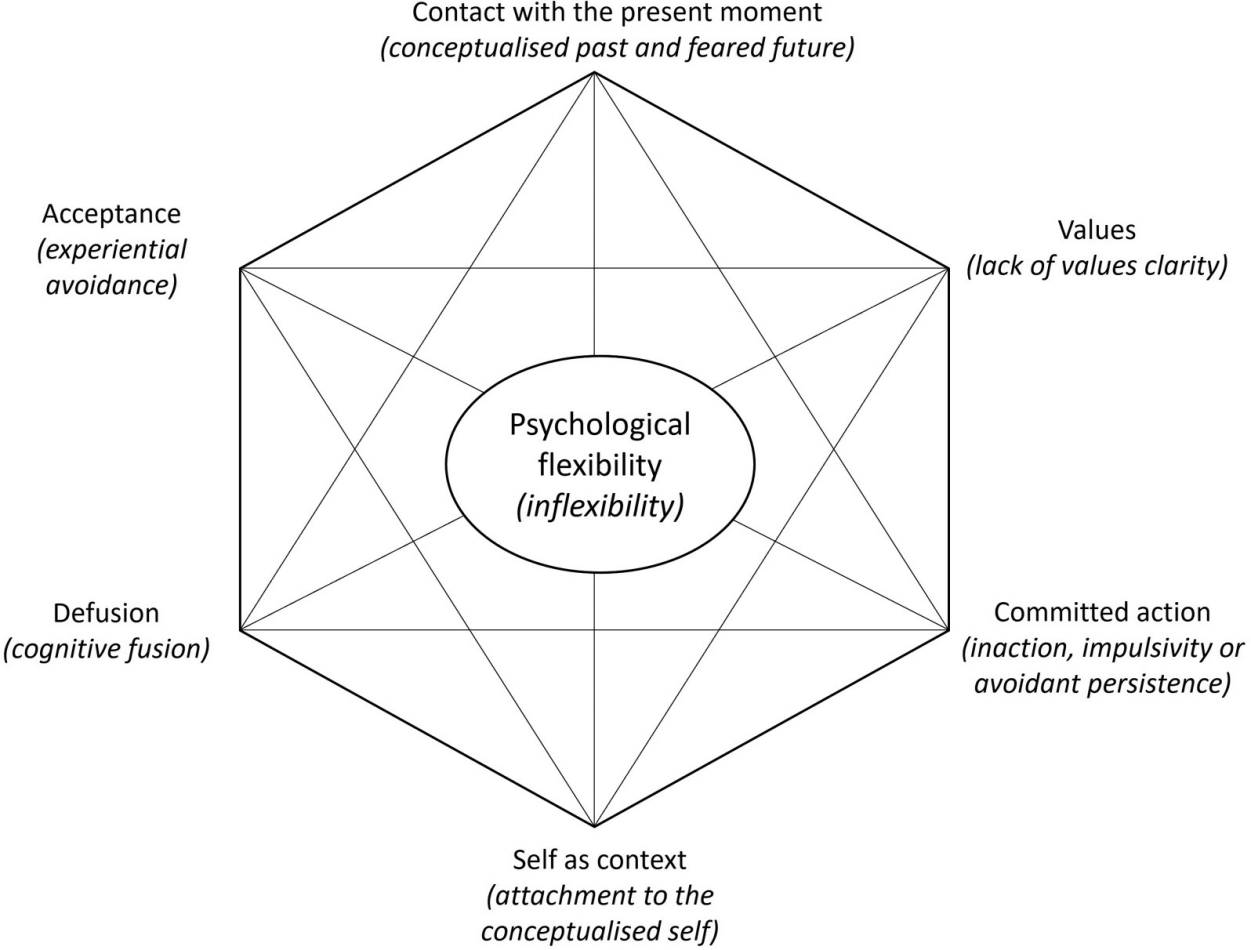
The acceptance and commitment therapy hexaflex (and in-hexaflex)^15^ (*adapted from copyright Steven C. Hayes. Used by permission*).

The process of change in Acceptance and Commitment Therapy is increased psychological flexibility^4,14,15^; ‘…to respond to situations in ways that facilitate valued goal pursuit’.^16^ Psychological flexibility is conceptualised with six facets that support or undermine its expression; the ‘Acceptance and Commitment Therapy hexaflex’^17^ and ‘in-hexaflex’^15^, respectively (see Figure 1). A growing number of tools purport to measure this mechanism of change.

Increasing psychological flexibility is posited to benefit outcomes such as depression and anxiety.^14,15^ Clinical trials seek standardised outcome measures to enable data pooling to guide clinical practice.^18^ The Core Outcome Measures in Effectiveness Trials (COMET) initiative supports standardisation, providing a taxonomy^19^ to classify outcomes used in trials. This may be useful in categorising measurement tools across different study types.

This review identified and summarised process and health-related outcome measures used in Acceptance and Commitment Therapy intervention studies with adult neurological populations, to inform the choice of measures for future studies to meet mental health needs and support well-being.^20^

### Objectives

1. Identify acquired neurological populations in which Acceptance and Commitment Therapy has been evaluated
2. Identify time-points at which the measurement tools were used
3. Identify and categorise tools used to explore Acceptance and Commitment Therapy process of change
4. Identify and categorise tools used to investigate health-related outcomes
5. Code outcome measurement tools according to COMET taxonomy^19^

## Methods

This review was informed by Arksey & O’Malley's five-stage scoping review methodology^20^ and enhanced using strategies recommended by Levac et al.^21^ Reporting was guided by the Preferred Reporting Items for Systematic reviews and Meta-Analyses extension for Scoping Reviews.^22^ A protocol for this review was written in advance of data collection and retrospectively published as a pre-print at: https://osf.io/cm4kt/.

**Eligibility criteria:** Studies were included in the review according to the following criteria:

Adult population (≥18 years old) with an acquired neurological condition. Our working definition of this term was:

- Acquired: not inherited, present at birth or neurodevelopmental.
- Neurological conditions: disorders of the brain, spinal column or peripheral nerves with a range of causes,^23^ including progressive conditions, such as MS and dementia, and acquired brain injuries, such as stroke and TBI.

Interventions of interest were those identified by the original authors as: Acceptance and Commitment Therapy, based on Acceptance and Commitment Therapy or where Acceptance and Commitment Therapy is a component (i.e. interventions were included if they used other strategies as well as Acceptance and Commitment Therapy), where intervention was provided due to the presence of the acquired neurological condition. We included studies with or without a comparator.

Included studies had at least one health-related outcome or Acceptance and Commitment Therapy-related process measure. The latter are those relevant to the mechanism of change in Acceptance and Commitment Therapy – psychological flexibility, or its facets (see Figure 1). Health-related outcome measures are those targeting any aspect of health (physical or mental). Measures that exclusively explored satisfaction, adherence, usability and cost were excluded.

We included all study designs that use pre- and post-measurement of outcomes and/or processes of change, for example, clinical reports, service evaluations, case studies, quasi-experimental studies and randomised controlled trials (RCTs) and excluded reviews. We included studies with full texts available in English. Some studies used translated versions of measures published in English. The translations are reported together with the English versions and not classified separately.

**Study identification and selection:** We searched the following bibliographic databases in 2020 and most recently on 29 June 2022: MEDLINE, PsycInfo and the Cumulative Index to Nursing and Allied Health Literature (CINAHL). As scoping reviews aim to be comprehensive, further information sources were consulted. The Association for Contextual Behavioural Science hosts a list; ‘Acceptance and Commitment Therapy Randomized Controlled Trials Since 1986’ (https://contextualscience.org/ACT_Randomized_Controlled_Trials) which we screened on 6 July 2022. *The Neuropsychologist* (a professional publication by the British Psychological Society, BPS) is not included in database searches, but as we were aware of a relevant article we screened all twelve published volumes for relevant studies.

Forward and backward searching was carried out with all included papers. Study authors were contacted in instances where the full paper was not available online, where additional information was required to make decisions about inclusion, or to answer methodological questions.

The umbrella term ‘acquired neurological conditions’ is not consistently used in the literature. Therefore, conditions were entered individually as keyword search terms and related MeSH terms, using the same terms as a Cochrane review with a similar population.^24^ The following search terms were used to capture the intervention [“Acceptance and Commitment Therapy”/] and “Acceptance and Commitment Therapy”.mp. The search strategies were drafted in consultation with a university librarian (See Supplemental materials for MEDLINE search strategy. This was adjusted for PsycInfo and CINAHL, using differing MeSH terms.).

Identified papers were imported into Endnote and duplicates removed. Screening comprised of two stages: title and abstract, full text.

Stage one – Title and abstract phase

- Initial learning phase – two members of the research team screened a small batch of papers (*n* = 5) and then discussed any discrepancies in screening decisions. This learning process was repeated a number of times until consistency was reached.
- Fifty percent of the papers were independently screened by two researchers. A moderate level of agreement (i.e. the value of kappa is ≥ 0.41 (Altman, 1991 cited in^25^) was required to proceed to the next stage. We erred on the side of inclusion if there was any disagreement between the researchers.
- The other 50% of total papers were screened by one researcher.

Stage two – Full text screening phase:

- As above, however disagreements were resolved by:
  - Discussion between the researchers aiming for consensus
  - Contacting the study authors to request additional information
  - Consensus discussion with all authors of this review (AB, SC, EP).

**Data extraction**: This was completed for papers that met the inclusion criteria. This included descriptive data such as: author, title, overall aim of the study, date, country, type of study, sample size, participants’ diagnoses. A bespoke data extraction tool was developed iteratively and piloted on a small number of studies (see Supplemental materials for a copy of this tool). All tools measuring processes or health-related outcomes were also extracted. For each tool, we extracted the name, the authors’ description of what the tool was measuring and the time points at which it was administered. Time points were converted into months post-intervention, subtracting the length of the treatment phase from the length of time post-baseline. A second researcher carried out data extraction for a randomly allocated third of the papers. The data extraction of the two researchers was compared and any inconsistences discussed in order to reach consensus.

**Data synthesis:** Study type was categorised based on author description and consensus between review authors. Measures were identified as *process measures* if they measured a mechanism of change, relevant to Acceptance and Commitment Therapy, as determined by the review authors. Acceptance and Commitment Therapy-specific measures were identified from the ACBS website and relevant Acceptance and Commitment Therapy literature.^14,26,27^

When measures were relevant to Acceptance and Commitment Therapy mechanisms but were not directly developed in the context of Acceptance and Commitment Therapy, information about each measure was reviewed (e.g. tool development papers), alongside data extracted from the studies. Consensus on whether to include the measure as a process measure was reached through discussion between review authors.

The process measurement tools identified were grouped according to what aspect of the Acceptance and Commitment Therapy hexaflex they measured (see Figure 1). The authors organised measures according to the hexaflex in a best-fit manner, with reference to rationale for the use of the measure provided in the included studies, papers describing the development of the measures, consulting a previous review,^28^ and discussion between authors of this review.

In addition, *health-related outcome measures* used in any of the studies were identified and:

1. Categorised broadly according to what they were measuring, based on data extracted from the papers, available development papers, assessment manuals and publisher descriptions, followed by consultation between the review authors. Then;
2. Coded according to the COMET taxonomy, which is organised into five core areas: death, physiological/clinical, life impact, resource use and adverse events. Each core area consists of a number of outcome domains. There are 38 outcome domains in total. As per guidance,^19^ each measure was coded according to all relevant outcome domains addressed in the measure; achieved through reviewing every item on each measure. If measures were not freely available to review individual items, coding was done based on overall aims and any other information freely available.

In order to ensure robustness of COMET coding, Susanna Dodd (author of the taxonomy) was consulted with questions on the coding process and regarding uncertainties in classification of specific tools. Furthermore, MP independently carried out COMET coding for 10% of the measures found. MP and HF first discussed any discrepancies and then Dodd was consulted regarding any outstanding uncertainties.

Step (2) was only carried out for measures extracted from studies identified in the original 2020 search. For reasons detailed in the discussion, this step was not completed for measures identified during the 2022 updated search.

## Results

**Figure 2.**
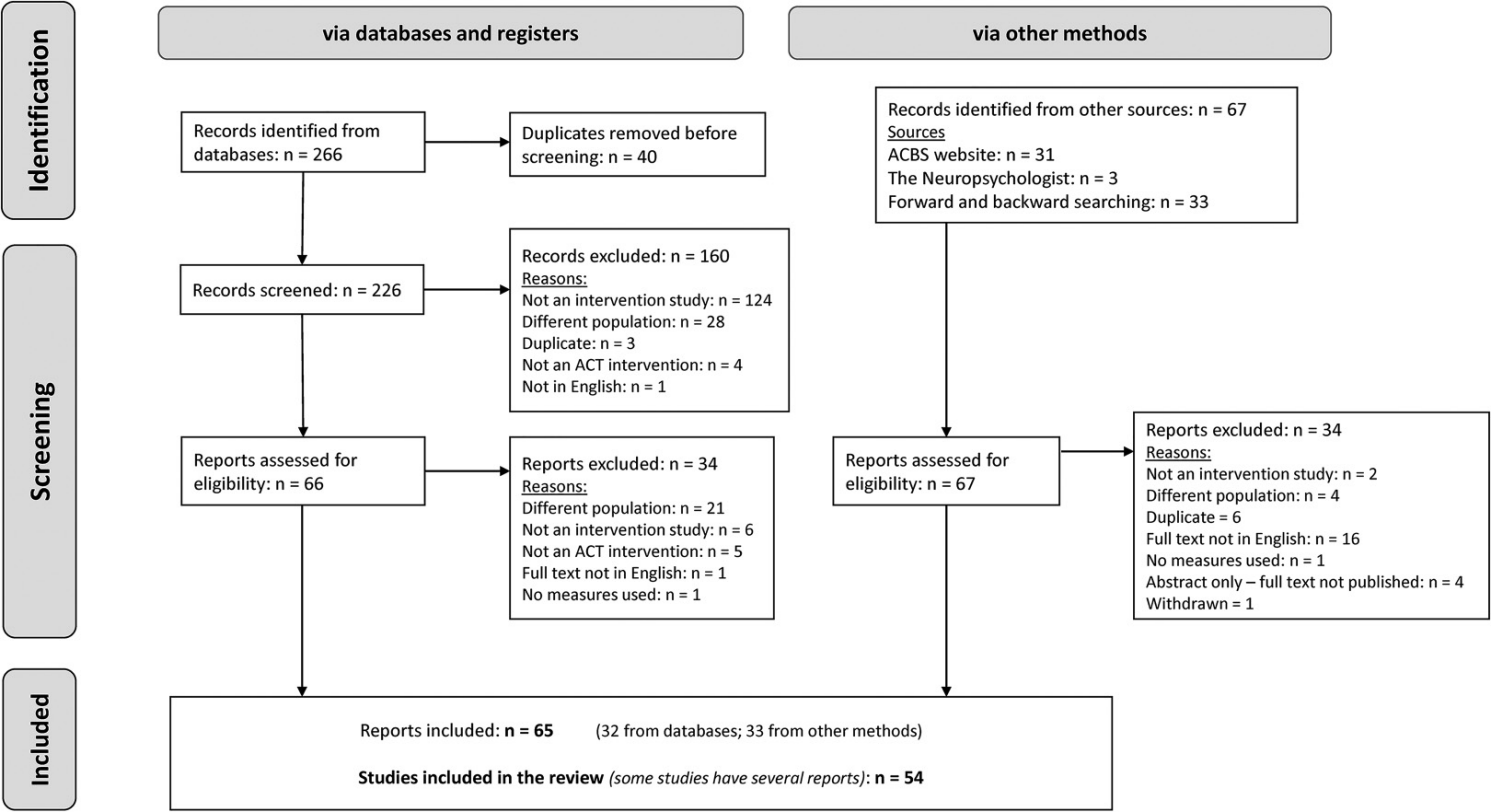
PRISMA flowchart – selection of sources of evidence.

Searching yielded 333 papers (see Figure 2). After duplicate removal and screening there were 54 included studies (from 65 reports). Reviewers had at least a substantial level of agreement (kappa = 0.67) at first stage screening.

Most of the studies were RCTs (*n* = 22) or other non-randomised research studies (i.e. those with non-randomised group allocation) (*n* = 16), with fewer clinical reports (*n* = 8) and *n* = 8 unclear for categorisation. The overall mean sample size was 44.1 (range 1–240). Most studies are from the UK (*n* = 13), Iran (*n* = 12), US (*n* = 9) and Australia (*n* = 7). In total, 136 measurement tools were extracted. Many studies did not specify whether tools used were selected on the basis of measuring processes of change or outcomes.

### Objective 1. Neurological populations

The participants had a range of acquired neurological conditions: of the 22 RCTs identified, eight were in MS, six in TBI, three in stroke, two in epilepsy, one in Parkinson's disease, one in spinal cord injury and one for both TBI and stroke. Some studies included participants with a range of different neurological conditions. Throughout the results, these studies are referred to as including ‘multiple conditions’.

The most commonly stated aim of studies was to reduce psychological distress (including anxiety, depression, post-traumatic stress disorder, stress, and emotional and psychological difficulties) (*n* = 30 studies). This aim was stated across studies in all neurological populations included in this review (apart from studies with multiple conditions). Physical symptoms were targeted in studies in certain conditions, for example, seizures in epilepsy (*n* = 2) and pain in MS (*n* = 3). Other studies had nuanced intended aims or outcomes for their interventions, for example, increasing psychological adjustment across multiple conditions, including TBI and MS (*n* = 5) and increasing resilience in MS (*n* = 5).

### Objective 2. Time points

All studies used pre- and post-intervention measures. Thirty-three of 54 studies (61%) carried out assessments at other follow-up time points, ranging from 1 month post-intervention to 12 months post-intervention.

### Objective 3. Process measurement tools

There were 32 different Acceptance and Commitment Therapy-related process measurement tools identified, across 38 of the 54 included studies (70%) (see Table 1). Eleven of the 24 tools (34%) were used in more than one study. Twenty-seven tools were generic and five were condition-specific. Two pain specific measures were identified and were used with MS populations.

**Table 1.** Summary of measures relevant to acceptance and commitment therapy processes.

Name of measurement tool	Target neurological population	*n* = studies that have used this tool	Conditions represented in the studies using this tool (study reference/s)
**Psychological flexibility (*n* = 29 studies)**			
Acceptance and Action Questionnaire: AAQ-II^29^^a^ or AAQ-9^30^^a^	General	21	Multiple sclerosis^8,10,11,31,35^ Stroke^36,37^ Stroke and TBI^38,39^ TBI^12,13,40,41^ Multiple conditions^42,43^ Brain tumour,^44^ Spinal cord injury^45^
Acceptance and Action Questionnaire-Acquired Brain Injury (AAQ-ABI)^46^^a^	Acquired brain injury	7	Stroke and TBI^38,39^ TBI^12,41,47^ Multiple conditions^48,49^
Chronic pain acceptance questionnaire (CPAQ)^50^^a^	General (chronic pain)	2	Multiple sclerosis^31,51^
The comprehensive assessment of Acceptance and Commitment Therapy processes (CompACT)^52^^a^	General	3	Multiple sclerosis^8,33,53^
Acceptance and Action Epilepsy Questionnaire (AAEpQ)^54^^a^	Epilepsy	1	Epilepsy^54^
Avoidance and Fusion Questionnaire-Youth (AFQ-Y)^55^^a^	General	1	Multiple conditions^48^
Adult Hope Scale^56^^b^	General	1	Stroke^57^
Adult State Hope Scale^58^^b^	General	1	Stroke^37^
Multidimensional Psychological Flexibility Inventory (MPFI)^59^	General	1	Multiple sclerosis^60^
**Values (12 studies)**			
Engaged Living Scale (ELS)^61^ *(also targeting committed action)*	General	1	TBI^47^
Valued Living Questionnaire (VLQ)^62^^a^	General	7	Multiple conditions^49^ Stroke and TBI^38,39^ TBI^63^ Multiple Sclerosis^8,33,64^
Survey of Life Principles Version 2.2 – Card sorting task^65^^a^	General	2	TBI ^12,47^
Values Bull's eye ^66^^a^	General	2	Epilepsy ^54^ TBI^67^
Valuing Questionnaire (VQ)^68^^a^	General	1	Multiple conditions = ^42^
**Defusion/fusion (10 studies)**			
Cognitive Fusion Questionnaire (CFQ)^69^^a^	General	6	Stroke and TBI^38,39^ TBI^63^ Multiple sclerosis^34,35^ Spinal cord injury^45^
Drexel Defusion Scale (DDS)^70^^a^	General	3	Multiple sclerosis^8,33,64^
Pain catastrophizing scale (PCS)^71^^b^	General (pain)	1	Multiple sclerosis^51^
**Acceptance/experiential avoidance (6 studies)**			
Acceptance of Chronic Health Conditions, MS version (ACHC-MS)^72^^b^	Multiple sclerosis	1	Multiple sclerosis^9^
Multiple Sclerosis Acceptance Questionnaire (MSAQ)^73^^a^	Multiple sclerosis	1	Multiple sclerosis^64^
Intolerance of Uncertainty Scale (IUS)^74^^b^	General	1	Multiple sclerosis^9^
White Bear Suppression Inventory (WBSI)^75^^b^	General	1	Multiple sclerosis^76^
Appraisal of Threat and Avoidance Questionnaire (ATAQ)^77^^b^	General	1	Multiple conditions^48^
Avoidance-Endurance Questionnaire Pain-Related Behavioral Responses Scale (AEQ)^78^^b^	General	1	Multiple sclerosis^51^
Emotional Avoidance Strategy Inventory (EAS)^79^	General	1	Multiple sclerosis^80^
**Contact with the present moment (6 studies)**			
Mindful Attention Awareness Scale (MAAS)^81^^b^	General	5	Multiple sclerosis^8,33,64,76^ Spinal cord injury^45^
Five Facet Mindfulness Questionnaire (FFMQ)^82^^b^	General	1	Multiple conditions^42^
**Committed action (2 studies)**			
Motivation for Traumatic Brain Injury Rehabilitation Questionnaire (MOT-Q)^83^) ^b^	Traumatic brain injury	2	TBI^12,41^
**Other (3 studies)**			
Cognitive Emotion Regulation Questionnaire (CERQ) ^b^^84^	General	1	Epilepsy^85^
Cognitive Flexibility Inventory (CFI) ^b^^86^	General	1	Spinal cord injury^87^
Emotion Regulation Questionnaire (ERQ) ^b^^88^	General	1	Spinal cord injury ^87^
Locus of Control Scale (LoC) ^b^^89^	General	1	Multiple sclerosis^90^
Sense of Coherence Scale (SOC) ^b^^91^	General	1	Multiple sclerosis ^90^

^a^ Developed with Acceptance and Commitment Therapy in mind.

^b^ Not developed specifically for Acceptance and Commitment Therapy.

Acceptance and Commitment Therapy-related processes were measured in studies of MS, acquired brain injury (including stroke and TBI), spinal cord injury, epilepsy, brain tumour and multiple conditions.

Composite measures of psychological flexibility were most commonly used, that is, in 29/38 (76%) of the studies that included a process measure. The Acceptance and Action Questionnaire^29,30^ was the tool most often used (21 studies, 55% of the studies including a process measure). A number of condition-specific variations of this measure were also identified in this review (Acceptance and Action Questionnaire-Acquired Brain Injury,^46^ Acceptance and Action Epilepsy Questionnaire,^54^ Chronic Pain Acceptance Questionnaire^50^ bringing the total number of studies using the Acceptance and Action Questionnaire and/or variants to 26.

Other tools measured a specific facet of the hexaflex (Figure 1). Values were measured most commonly (and most often using the Valued Living Questionnaire,^62^*n* = 7 studies), followed by defusion (or conversely, cognitive fusion), acceptance (or conversely experiential avoidance), contact with the present moment and committed action. No tools specifically targeted self-as-context. In contrast, five other process measures were identified that were not specifically linked to any of the hexaflex facets.

### Objective 4. Outcome measurement tools

There were 104 distinct outcome measurement tools extracted from the 54 included studies. Seventy-three (70%) of these tools were used once. Table 2 lists all the extracted tools organised by category, with COMET coding presented for tools extracted from studies identified in the 2020 search (see objective 5 for a summary of this COMET coding).

**Table 2.** Outcome measurement tools.

Name of measurement tool	Target neurological population	*n* = studies that have used this tool	Conditions represented in studies using this tool [study reference]	COMET outcome domains
**Health status, quality of life and well-being (*n* = 27 studies)**				
12-Item Short Form Survey (SF-12)^92^	General a	6	Stroke and TBI^38,39^ TBI^12,40,41^ Epilepsy^93^	Emotional, general, perceived health status, physical, role, social
EQ-5D (5L specified in 3) ^94^	General a	6	Stroke^37,57^ Stroke and TBI^38^ Multiple sclerosis^11,53,60^	Emotional, general, perceived health status, physical, psychiatric, role
Multiple Sclerosis Quality of Life Instrument (MSQoL-54)^95^	Multiple sclerosis	4	Multiple sclerosis^8,33,60,64^	Cognitive, emotional, global quality of life, nervous system, perceived health status, physical, role, social
Satisfaction with Life Scale (SWLS)^96^	General a	4	Epilepsy^97,98^ TBI^47^ Multiple conditions^49^	Global quality of life
World Health Organization Quality Of Life (WHOQOL-BREF)^99^	General a	4	TBI^40^ Epilepsy^97,98^ Spinal cord injury^45^	Cognitive, emotional, global quality of life, perceived health status, personal circumstances, physical, role, social
Multiple Sclerosis Impact Scale (MSIS-29)^100^	Multiple sclerosis	3	Multiple sclerosis^11,53,101^	Cognitive, emotional, physical, role, social
Warwick and Edinburgh Mental Well-being Scale^102^	General a	2	Stroke^57^ Multiple conditions^49^	Cognitive, emotional, social
EuroQol Visual Analogue Scale (EQ-VAS)^94^	General a	1	Multiple conditions^42^	Perceived health status
Functional Assessment of Cancer Therapy scale – Brain^103^	Cancer – brain	1	Brain tumour^44^	Adverse events/effects, cognitive, emotional, general, global quality of life, nervous system physical, role, social
Mental Health Continuum Short Form (MHC-SF)^104^	General a	1	Multiple sclerosis^60^	
Multiple Sclerosis Quality of life index (QLI)^105^	Multiple sclerosis	1	Multiple sclerosis^31^	Cognitive, delivery of care, emotional, global quality of life, perceived health status, personal circumstances, physical, role social
Numerical rating scale ‘Which numeric rating score from 1 (very bad) to 10 (very well) do you give your life in general?’ (study specific)	General	1	Multiple sclerosis^34^	
Parkinson's Disease Questionnaire-39 (PDQ-39)^106^	Parkinson's disease	1	Parkinson's disease^107^	Cognitive, emotional, nervous system, physical, social
Personal Well-being Index (PWI)^108^	General a	1	Epilepsy^54^	Emotional, global quality of life, perceived health status, social
Quality of life after brain injury (QOLIBRI)^109^	TBI	1	TBI^47^	
Quality of Life Inventory (QOLI)^110^	General a	1	Multiple sclerosis^76^	Emotional, global quality of life, social, role
Quality of life rating on a scale of 1–10 (study specific)	General a	1	TBI^63^	Global quality of life
Sheehan Disability Scale^111^	General a	1	TBI^40^	Role, social
Schedule for the Evaluation of Individual Quality of Life-Direct Weighting SEIQoL-DW^112^	General a	1	Multiple sclerosis^8^	Outcome domains depend on individual choices
Short Form 36 Health Survey (SF-36)^113^	General a	1	Multiple sclerosis^76^	Emotional, general, perceived health status, physical, role, social
Spinal Cord Injury – Quality of Life (SCI-QOL)^114^	Spinal cord injury	1	Spinal cord injury^45^	
Visual analogue scale – ‘How content are you with your daily life?’ (study specific)	General	1	Multiple sclerosis^34^	
Wellbeing Evaluation Scale (WES)^115^	General a	1	Multiple conditions^116^	Emotional, physical, social
**Anxiety and depression (*n* = 22 studies)**				
Hospital Anxiety and Depression Scale (HADS)^117^	General a	14	Multiple sclerosis^8,10,32,33,53,60^ Multiple conditions^49,118^ Stroke and TBI^38,39^ Stroke^37^ TBI^12,41,63^	Emotional, physical, psychiatric
Depression, Anxiety, and Stress Scale (DASS-21)^119^	General a	9	Stroke^120^ Stroke and TBI^38,39^ TBI^12,41,47,67^ Multiple sclerosis^121^; Spinal cord injury^45^	Emotional, psychiatric
Brief Symptom Inventory – 18 (BSI-18)^122^	General a	3	TBI^13,40^	Cognitive, emotional, psychiatric, social
Seven-point Likert scale – ‘I feel depressed’ and ‘I feel anxious’ (study specific)	General	1	Stroke and TBI^39^	
**Depression (*n* = 13 studies)**				
Beck Depression Inventory (BDI I or II)^123^	General a	6	Brain tumour^44^ Multiple sclerosis^10,76,124,125^ Parkinson's disease^107^	Emotional, physical, psychiatric, role, social
Patient Health Questionnaire (PHQ-9)^126^	General a	4	Multiple conditions^43^ Multiple sclerosis^11^ Stroke^57^TBI^40^	Cognitive, emotional, physical, psychiatric
Neurological Disorders Depression Inventory for Epilepsy (NDDI-E)^127^	Epilepsy	1	Epilepsy^93^	Emotional, psychiatric
Hamilton Depression Scale (HAMD-24)^128^	General a	1	Stroke^129^	
Self-rating Depression Scale (SDS)^130^	General a	1	Multiple Sclerosis^35^	
**Anxiety (*n* = 10 studies)**				
Generalised Anxiety Disorder Assessment (GAD-7)^131^	General	5	Multiple conditions^43^ Multiple sclerosis^9,11^ Stroke^57^ Epilepsy^93^	Emotional, psychiatric
Beck Anxiety Inventory (BAI)^132^	General a	2	Parkinson's disease^107^ Multiple sclerosis^124^	Emotional, psychiatric
Hamilton Anxiety Rating Scale^133^	General a	1	Stroke^134^	Cognitive, emotional, psychiatric
State-Trait Anxiety Inventory (STAI)^135^	General a	1	Brain tumour^44^	Emotional, psychiatric
Self-rating Anxiety Scale (SAS)^136^	General a	1	Multiple sclerosis^35^	
**Participation (*n* = 13 studies)**				
Utrecht Scale for Evaluation of Rehabilitation-Participation (USER-P)^137^	General a, various b	3	Stroke and TBI^38,39^ Multiple sclerosis^34^	Physical, role, social
Barthel Index (BI)^138^	Various b	2	Multiple sclerosis^139^ Stroke^129^	
Participation Assessment with Recombined Tools-Objective (PART-O)^140^	TBI	2	TBI^13^	Physical, role, social
Sydney Psychosocial Reintegration Scale-2 (SPRS-2)^141^	ABI	2	TBI^12,41^	Physical, role, social
Community Integration Questionnaire (CIQ)^142^	TBI	1	Multiple conditions^49^	
Nottingham Extended Activities of Daily Living index (NEADL)^143^	Stroke	1	Parkinson's disease^107^	Physical, social
Participation Objective, Participation Subjective (POPS)^144^	Brain injury	1	Multiple conditions^48^	Physical, role, social
Work and Social Adjustment Scale (WSAS)^145^	General a	1	Epilepsy^93^	Physical, role, social
**Psychological disorders – general (*n* = 6 studies)**				
General Health Questionnaire-12 (GHQ-12)^146^	General a	2	TBI^12,41^	Cognitive, emotional, psychiatric
Symptom Checklist-90-Revised (SCL-90-R)^147^	General a	2	Multiple conditions^48^ TBI^148^	Emotional, psychiatric
Clinical Outcome in Routine Evaluation-10 (CORE-10)^149^	General a	1	Multiple conditions^42^	Emotional, psychiatric, social
Structured Clinical Interview for DSM-5 (SCID-5)^150^	General a	1	Brain tumour^44^	Emotional, psychiatric
**Stress (*n* = 6 studies)**				
Perceived Stress Scale (PSS)^151^	General a	4	Multiple sclerosis^8,33,152^ , multiple conditions^118^	Emotional
Perceived Stress Questionnaire (PSQ)^153^	General a	1	Epilepsy^154^	
Additional stress since last assessment – dichotomous question (study specific)	General a	1	Brain tumour^44^	Emotional
**Resilience (*n* = 5 studies)**				
Connor-Davidson Resilience Scale (CD-RISC 25)^155^	General a	4	Multiple sclerosis^8,33,60,101^	Cognitive, emotional, social
15-item Resilience Scale (RS-15)^156^	General a	1	Multiple sclerosis^64^	Cognitive, emotional
**Pain (*n* = 4 studies)**				
Brief Pain Inventory (BPI)^157^	General a	2	Multiple sclerosis^31,51^	General
MOS Pain Effects Scale (PES)^158^	Multiple sclerosis	2	Multiple sclerosis^31,76^	Emotional, physical, role,
McGill Pain Questionnaire (MGPQ)^159^	General a	1	Multiple sclerosis^160^	General
**Fatigue (*n* = 3 studies)**				
Modified Fatigue Impact Scale (MFIS)^158^	Modified Fatigue Impact Scale (MFIS)^158^	Modified Fatigue Impact Scale (MFIS)^158^	Modified Fatigue Impact Scale (MFIS)^158^	Modified Fatigue Impact Scale (MFIS)^158^
Fatigue Severity Scale (FSS)^161^	Fatigue Severity Scale (FSS)^161^	Fatigue Severity Scale (FSS)^161^	Fatigue Severity Scale (FSS)^161^	Fatigue Severity Scale (FSS)^161^
Multidimensional Fatigue Inventory (MFI)^162^	Multidimensional Fatigue Inventory (MFI)^162^	Multidimensional Fatigue Inventory (MFI)^162^	Multidimensional Fatigue Inventory (MFI)^162^	Multidimensional Fatigue Inventory (MFI)^162^
**Post-traumatic stress disorder (*n* = 3 studies)**				
PTSD Checklist, Military Version^163^	General a	1	TBI^40^	Cognitive, emotional, physical psychiatric, social
Impact of events scale revised (IES-R)^164^	General a	1	TBI^63^	Cognitive, emotional, psychiatric
Clinician Administered Post-Traumatic Stress Disorder Scale (CAPS)^165^	General a	1	Spinal cord injury^166^	
PTSD symptom scale – interview for diagnostic statistical manual DSM-5^167^	General a	1	TBI^63^	Cognitive, emotional, psychiatric
**Seizures (*n* = 3 studies)**				
Seizure frequency (study specific)	Epilepsy	3	Epilepsy^93,97,98^	Nervous system
Seizure index (frequency × duration) (study specific)	Epilepsy	2	Epilepsy^97,98^	Nervous system
**Self-efficacy (*n* = 3 studies)**				
General Self-Efficacy Scale (GSES)^168^	General a	1	Parkinson's disease^107^	Cognitive, emotional
Multiple sclerosis Self Efficacy Scale (MSSE)^169^	Multiple sclerosis	1	Multiple sclerosis^53^	
TBI Self-Efficacy Scale (TBI-SES)^170^	TBI	1	Multiple conditions^49^	
**Sleep (*n* = 3 studies)**				
Athens Insomnia Scale (AIS)^171^	General a	1	Multiple sclerosis^35^	
Insomnia Severity Index^172^	General a	1	TBI^40^	Cognitive, emotional, physical, psychiatric, role
Pittsburgh Sleep Quality Index (PSQI)^173^	General a	1	Multiple sclerosis^35^	
Rating of sleep quality – poor to excellent (study specific)	General a	1	Brain tumour^44^	Psychiatric
**Memory (*n* = 2 studies)**				
Everyday Memory Questionnaire-Revised (EMQ-R)^174^	Various b	1	Multiple conditions^49^	
Wechsler Memory Scale – Third Edition (WMS-III)^175^		1	Multiple sclerosis^176^	
**Other outcomes (*n* = 20 studies)**				
Positive and Negative Affect Schedule (PANAS)^177^	General a	3	TBI^12,41^ Multiple sclerosis^60^	Emotional
Post Traumatic Growth Inventory^178^	General a	2	Stroke^37^ Multiple sclerosis^90^	Emotional, social
Self-Compassion Scale (SCS,^179^	General a	2	Multiple conditions^48^ Spinal cord injury^45^	Emotional
10 Meter Walk Test (10MWT)^180^	Various b	1	Parkinson's disease^107^	Physical
Alcohol Use Disorders Identification Test (AUDIT)^181^	General a	1	TBI^40^	Emotional, physical, psychiatric, role
Cognitive Failure Questionnaire (CFQ)^182^	General a	1	Multiple Sclerosis^80^	
Columbia Suicide Severity Rating Scale^183^	General a	1	TBI^40^	Emotional, physical, psychiatric
Computerized Stroop Test (CST)^184^		1	Multiple sclerosis^176^	
Death Attitude Profile-Revised (DAP-R)^185^	General a	1	Multiple sclerosis^139^	
Dimensions of Anger Reactions II^186^	General a	1	TBI^40^	Cognitive, emotional, social,
Engagement in Meaningful Activities Survey (EMAS)^187^	General a	1	Spinal cord injury^45^	
Freezing of Gait (FOG) Questionnaire^188^	Parkinson's disease	1	Parkinson's disease^107^	Nervous system, physical
Functional Independence Measure (FIM) Scale^189^	General a	1	Spinal cord injury^166^	
Key Behaviors Change Inventory (KBCI)^190^	Traumatic brain injury	1	TBI^148^	Cognitive, emotional, social
Mayo Portland Adaptability Inventory (MPAI-4)^191^	Acquired brain injury	1	Multiple conditions^48^	Cognitive, emotional, nervous system, physical, role, social
National Institutes of Health Stroke Scale (NIHSS)^192^	Stroke	1	Stroke^129^	
Orbach & Mikulincer Mental Pain Scale (OMMP)^193^	General a	1	Multiple sclerosis^194^	
Orientation Toward Productive Activities Scale^195^	General a	1	Multiple conditions^48^	Cognitive, emotional, physical, role, social
Paced Auditory Serial Addition Test (PASAT)^196^	Various b	1	TBI^148^	Cognitive
Psychological Adaptation Scale (PAS)^197^	General a	1	Multiple sclerosis^198^	Cognitive, emotional, social
Rivermead Postconcussion Symptoms Questionnaire (RPQ)^199^	Brain injury	1	TBI^200^	Cognitive, emotional, nervous system
Rosenberg Self-Esteem Scale (RSES)^201^	General a	1	Epilepsy^93^	Emotional
Schneider's life expectancy questionnaire	General a	1	Multiple sclerosis^202^	General
Social Phobia Inventory (SPIN)^203^	General a	1	Epilepsy^154^	
Templer Death Anxiety^204^	General a	1	Multiple sclerosis^205^	Emotional
Things I’d like to change (TILTC)^206^	General a	1	Multiple conditions^43^	Outcome domains depend on individual choices
Timed One Leg Stance Test (OLST)^207^	Various b	1	Parkinson's disease^107^	Physical
Wearing-off Questionnaire (WOQ-19)^208^	Parkinson's disease	1	Parkinson's disease^107^	Nervous system

^a^ Tools which are not related to a neurological condition.

^b^ Tools designed for use in neurological conditions of various causes.

The first category is **Health status, Quality of life and Well-being.** There were 23 tools identified, across 27 studies. Many tools measured a combination of health status, quality of life and well-being and are reported together in Table 2. All neurological conditions represented in this review included a measure in this category. Seven measures in this category were condition specific, to MS (*n* = 3), cancer – brain (*n* = 1), Parkinson's disease (*n* = 1), TBI (*n* = 1) and spinal cord injury (*n* = 1).

The most commonly used measurement tools were the 12-Item Short Form Survey^92^ (*n* = 6 studies) and the EQ-5D^94^ (*n* = 6 studies), followed by the Multiple Sclerosis Quality of Life Instrument,^95^ Satisfaction with Life Scale^96^ and the World Health Organization Quality Of Life measure^99^ (each used in n = 4 studies).

The second category is **anxiety and depression and other psychological disorders.** Overall, there were 22 tools identified that measured a psychological disorder, across 38 studies. Most commonly, these tools measured anxiety and/or depression and were used across each of the neurological conditions represented in this review. All measures in this category were general, apart from one epilepsy specific measure. Measurement tools assessing both anxiety and depression were used in 22 studies, for example, the Hospital Anxiety and Depression Scale^117^ (*n* = 14 studies), and the Depression, Anxiety, and Stress Scale^119^ (*n* = 9 studies). Tools measuring depression alone were used in 13 studies. The most commonly used tools were the Beck Depression Inventory^123^ (*n* = 6 studies) and Patient Health Questionnaire^126^ (*n* = 4 studies). Anxiety alone was measured in 10 studies, for example, the Generalised Anxiety Disorder Assessment^131^ in five studies and the Beck Anxiety Inventory^132^ in two. The other tools identified were general measures of psychological disorders (*n* = 6 studies) and measures of post-traumatic stress disorder (*n* = 3 studies). Post-traumatic stress disorder was only measured in studies in traumatic brain and spinal cord injury. Twenty-two of the studies included both a measure of health status, quality of life, well-being and a measure of a psychological disorder/s.

Further outcome categories identified were participation (i.e. involvement in life situations) (*n* = 13 studies), stress (*n* = 6 studies), resilience (*n* = 5 studies), pain (*n* = 4 studies), fatigue (*n* = 3 studies), seizures (*n* = 3 studies), self-efficacy (*n* = 3), sleep (*n* = 3 studies) and memory (*n* = 2 studies). Each tool in these categories was used in a maximum of four studies. Pain, fatigue and resilience were only measured in MS and seizures were specific to studies in epilepsy.

### Objective 5. COMET taxonomy

Fifty-three of 76 tools (those identified in the 2020 search) were freely available and reviewed item-by-item. COMET coding of all extracted measures showed that three of the five COMET core areas (physiological/clinical, life impact and adverse events) and 13 of the 38 outcome domains were represented in the data set (see Table 3). As discussed in methods, COMET outcome domains are not mutually exclusive (see Table 2).

**Table 3.** COMET core areas and outcome domains represented in the data set.

Core area	Outcome domain	Frequency
Physiological/clinical	Psychiatric	22
	General	10
	Nervous system	9
Life impact	Emotional functioning/wellbeing^a^	50
	Physical functioning^a^	32
	Role functioning^a^	22
	Social functioning^a^	13
	Cognitive functioning^a^	9
	Global quality of life	8
	Perceived health status	8
	Personal circumstances	2
	Delivery of care	1
Adverse events	Adverse events/effects	1

^a^ The names of these domains are shortened to physical, social, role, emotional and cognitive in Table 2.

The physiological/clinical core areas present in the data are in line with the eligibility criteria for this review. Within the life impact and adverse event core areas, all outcome domains were present in the data. The core areas not represented are death and resource use, ineligible for this review. The most frequently occurring core areas and domains are summarised below.

The most commonly occurring physiological domain was ‘psychiatric’. There were also ‘general’ outcomes, including pain, fatigue and life expectancy. According to the COMET guidance, the physiological/clinical domains should be classified according to underlying cause/body system. Therefore, certain measures relating to neurological conditions specifically were coded under the ‘nervous system’ domain (e.g. Parkinson's Disease Questionnaire-39^106^ and Functional Assessment of Cancer Therapy scale – Brain.^103^).

The most commonly classified domain was the life impact domain ‘emotional functioning/well-being’. Measures of mental health signs and symptoms (e.g. anxiety and depression) were classified in this domain, as well as under ‘psychiatric’ (above).

The health status, quality of life and well-being measures identified were coded against all the COMET core areas and outcome domains above (see Table 2 for measure specific results). As per taxonomy guidance, only composite items on quality of life or health status were classified under the ‘global quality of life’ and ‘perceived health status’ domains. For example, the World Health Organization Quality Of Life measure^99^ was classified as such, as it contains the composite questions ‘How would you rate your quality of life?’ and ‘How satisfied are you with your health?’. Most health status, quality of life and/or well-being measures included items targeting multiple individual domains. These frequently included the functioning domains (physical, social, role, emotional/well-being and cognitive) as well as often including an item/s about physiological signs and symptoms.

## Discussion

This review found that a large number of studies utilised Acceptance and Commitment Therapy for people with a range of acquired neurological conditions (with MS being the most common), using many different measurement tools. Measures targeting psychological flexibility as a composite were commonly used and, in accordance with previous research,^209^, this was most often measured by the Acceptance and Action Questionnaire-II.^29^ The majority of studies aimed to reduce psychological distress and thus selected a wide variety of non-condition specific health-related outcome measures exploring distress, anxiety and/or depression.

This proliferation of measures warns us of challenges pooling and comparing data unless we reach consensus on process and outcome measures for future studies. We found that the most commonly measured COMET taxonomy domains were in the life impact core area. This is encouraging as the theoretical model of Acceptance and Commitment Therapy aims to improve functioning and well-being, rather than just focusing on the reduction of psychological distress.^7,18^ The majority of studies reported measurement time points relative to the end of the intervention in contrast to recommendations to report relative to baseline.^210^ In categorising the measures it became clear that there is inconsistency in the definitions and use of terms such as health status, quality of life and well-being, as previously reported.^211^

A strength of this review is the meticulous process of categorising and coding all measures with reference to literature, including the novel application of the COMET taxonomy to Acceptance and Commitment Therapy research.^19^ Coding consensus was achieved through substantial consultation both internally (paper authors) and externally (with Susanna Dodd, author of the COMET taxonomy). Item-by-item coding of each health-related outcome measure (when freely available) enabled comprehensive mapping according to all outcome domains measured. However, a risk of item-by-item classification is that it does not take into account how the measurement tools have been constructed, and therefore may overestimate the domains that have been measured. Furthermore, as the domains in the COMET taxonomy are not mutually exclusive, the coding process did not aid the categorisation of the many tools into distinct groups. Due to these limitations, COMET categorisation was not completed for additional measures extracted when the review was updated in 2022.

A ‘broader’ level categorisation of the measures (as described in methods) was also provided to make overall sense of the tools. This broad categorisation of the outcome measures, and the categorisation of process measures according to the Acceptance and Commitment Therapy hexaflex, were done in a best-fit manner by the authors. Data extracted from the studies themselves were used to inform these decisions, but inconsistencies in this information, and lack of reporting, meant that author consultation was often used, which remains subjective and open to further debate.

This review is limited in only including studies reported in English. However, the inclusion of multiple study types, and of studies that included Acceptance and Commitment Therapy plus other interventions, enabled comprehensive identification of measures used in Acceptance and Commitment Therapy in acquired, neurological populations.

A strength of this review is the enhancement of the scoping review methodology^21^ through use of double reviewing during study selection, data extraction and data synthesis.

This review contributes recommendations and future research directions. Our findings highlight reporting inconsistencies in the field that could be improved. The use of suitable reporting guidance (https://www.equator-network.org/) would facilitate data synthesis from Acceptance and Commitment Therapy research trials in systematic reviews. Authors should clarify whether measures were selected to explore processes of change or health outcomes. Authors should explicitly state the process and outcome domains that they are aiming to measure, as well as the measurement tools themselves. There is on-going development of core outcome sets relevant to a number of the populations included in this review. Where available, it is recommended that clinical trials of Acceptance and Commitment Therapy use relevant core outcome sets to inform their choice of measures.

The findings of this review of what has been measured are a useful resource to support identification of candidate measurement tools. However, this cannot be extrapolated to inform what should be measured, or which tools should be used.

In order for specific recommendations to be made for use of Acceptance and Commitment Therapy in acquired, neurological populations, future research is required and should include consensus by experts, use of patient, carer and public involvement,^212,213^ and examination of the psychometric properties of the measures.

## Conclusion

This review summarises a detailed categorisation of the process and outcome measures previously used in Acceptance and Commitment Therapy studies in acquired neurological populations. Acceptance and Commitment Therapy has primarily been used to target psychological distress, but other outcomes including physical health outcomes have also been targeted. We highlight that a wide range of both process and outcome measurements are in use, with little guidance available on selection. This review provides a resource for other researchers and could support development of core outcome sets.

**Clinical messages**

- Mental health needs of adults with neurological conditions are poorly addressed and there is an imperative to deliver evidence-based interventions to promote well-being.
- Clinical guidance on whether Acceptance and Commitment Therapy is useful for this clinical population is being hampered by the lack of agreement on which of the many measures available should be used to evaluate the process of change and outcomes following intervention.
- Key stakeholders should be involved in consensus-based decision-making, which draws on resources such as this review of candidate measures.

## Supplemental Material

sj-docx-1-cre-10.1177_02692155221144554 - Supplemental material for A scoping review to identify process and outcome measures used in acceptance and commitment therapy research, with adults with acquired neurological conditions Click here for additional data file. Supplemental material, sj-docx-1-cre-10.1177_02692155221144554 for A scoping review to identify process and outcome measures used in acceptance and commitment therapy research, with adults with acquired neurological conditions by Hannah Foote, Audrey Bowen, Sarah Cotterill, Geoff Hill, Matilde Pieri and Emma Patchwood in Clinical Rehabilitation

sj-pdf-2-cre-10.1177_02692155221144554 - Supplemental material for A scoping review to identify process and outcome measures used in acceptance and commitment therapy research, with adults with acquired neurological conditions Click here for additional data file. Supplemental material, sj-pdf-2-cre-10.1177_02692155221144554 for A scoping review to identify process and outcome measures used in acceptance and commitment therapy research, with adults with acquired neurological conditions by Hannah Foote, Audrey Bowen, Sarah Cotterill, Geoff Hill, Matilde Pieri and Emma Patchwood in Clinical Rehabilitation
