# Supplementary material for: A scoping review to identify process and outcome measures used in acceptance and commitment therapy research, with adults with acquired neurological conditions
Source: Clin Rehabil. 2022 Dec 20;37(6):808–35. doi: 10.1177/02692155221144554 (PMC10134096; doi:10.1177/02692155221144554)
Supplement: sj-docx-1-cre-10.1177_02692155221144554 - Supplemental material for A scoping review to identify process and outcome measures used in acceptance and commitment therapy research, with adults with acquired neurological conditions [file sj-docx-1-cre-10.1177_02692155221144554.docx]

# Appendix 1

*MEDLINE search strategy*
Search Strategy:

| **#** | **Searches** |
| --- | --- |
| 1 | Cerebrovascular Disorders/ |
| 2 | Basal Ganglia Cerebrovascular Disease/ |
| 3 | Brain Ischemia/ |
| 4 | Carotid Artery Diseases/ |
| 5 | Cerebrovascular Trauma/ |
| 6 | Intracranial Arterial Diseases/ |
| 7 | "Intracranial Embolism and Thrombosis"/ |
| 8 | Intracranial Hemorrhages/ |
| 9 | Stroke/ |
| 10 | Brain Infarction/ |
| 11 | stroke.mp. |
| 12 | poststroke.mp. |
| 13 | post-stroke.mp. |
| 14 | cva.mp. |
| 15 | cerebrovascular accident.mp. |
| 16 | cerebrovascular insult.mp. |
| 17 | cerebral vascular accident.mp. |
| 18 | cerebral vascular insult.mp. |
| 19 | brain vertebrobasilar.mp. |
| 20 | cerebral subarachnoid.mp. |
| 21 | brain subarachnoid.mp. |
| 22 | trauma* brain injur*.mp. |
| 23 | acquired brain injur*.mp. |
| 24 | Brain Injuries/ |
| 25 | brain damage.mp. |
| 26 | Brain Concussion/ |
| 27 | brain hemorrhage.mp. |
| 28 | Brain Injury, Chronic/ |
| 29 | Cerebral Hemorrhage/ |
| 30 | Craniocerebral Trauma/ |
| 31 | Intracranial Hemorrhage, Traumatic/ |
| 32 | Brain Hemorrhage, Traumatic/ |
| 33 | Encephalitis/ |
| 34 | Meningitis, Viral/ |
| 35 | meningitis, viral.mp. |
| 36 | Encephalitis.mp. |
| 37 | Brain Abscess/ |
| 38 | Central Nervous System Infections/ |
| 39 | brain infection*.mp. |
| 40 | cerebral infection$.mp. |
| 41 | brain abscess.mp. |
| 42 | Multiple Sclerosis/ |
| 43 | cerebral ischemia.mp. |
| 44 | Parkinson Disease/ |
| 45 | Dementia/ |
| 46 | Epilepsy/ |
| 47 | Neurodegenerative Diseases/ |
| 48 | Brain Diseases/ |
| 49 | Alzheimer Disease/ |
| 50 | Motor Neuron Disease/ |
| 51 | Neuromuscular Diseases/ |
| 52 | Cognitive Dysfunction/ |
| 53 | "Acceptance and Commitment Therapy"/ |
| 54 | "Acceptance and Commitment Therapy".mp. |
| 55 | 53 or 54 |
| 56 | brain injury.mp. |
| 57 | motor neuron* disease.mp. |
| 58 | dementia.mp. |
| 59 | alzheimer.mp. |
| 60 | multiple sclerosis.mp. |
| 61 | parkinson* disease.mp. |
| 62 | epilepsy.mp. |
| 63 | Cognition Disorders/ |
| 64 | cognition disorders.mp. |
| 65 | head injury.mp. |
| 66 | Hematoma/ |
| 67 | hematoma.mp. |
| 68 | Cerebrovascular Disease.mp. |
| 69 | Hypoxia, Brain/ |
| 70 | hypoxia.mp. |
| 71 | Hypoxia-Ischemia, Brain/ |
| 72 | anoxia.mp. |
| 73 | Subarachnoid Hemorrhage/ |
| 74 | neuromuscular disease*.mp. |
| 75 | brain infarction.mp. |
| 76 | brain concussion.mp. |
| 77 | brain injur*.mp. |
| 78 | 1 or 2 or 3 or 4 or 5 or 6 or 7 or 8 or 9 or 10 or 11 or 12 or 13 or 14 or 15 or 16 or 17 or 18 or 19 or 20 or 21 or 22 or 23 or 24 or 25 or 26 or 27 or 28 or 29 or 30 or 31 or 32 or 33 or 34 or 35 or 36 or 37 or 38 or 39 or 40 or 41 or 42 or 43 or 44 or 45 or 46 or 47 or 48 or 49 or 50 or 51 or 52 or 56 or 57 or 58 or 59 or 60 or 61 or 62 or 63 or 64 or 65 or 66 or 67 or 68 or 69 or 70 or 71 or 72 or 73 or 74 or 75 or 76 or 77 |
| 79 | 55 and 78 |
| 80 | Cerebrovascular Disorders/ |
| 81 | Basal Ganglia Cerebrovascular Disease/ |
| 82 | Brain Ischemia/ |
| 83 | Carotid Artery Diseases/ |
| 84 | Cerebrovascular Trauma/ |
| 85 | Intracranial Arterial Diseases/ |
| 86 | "Intracranial Embolism and Thrombosis"/ |
| 87 | Intracranial Hemorrhages/ |
| 88 | Stroke/ |
| 89 | Brain Infarction/ |
| 90 | stroke.mp. |
| 91 | poststroke.mp. |
| 92 | post-stroke.mp. |
| 93 | cva.mp. |
| 94 | cerebrovascular accident.mp. |
| 95 | cerebrovascular insult.mp. |
| 96 | cerebral vascular accident.mp. |
| 97 | cerebral vascular insult.mp. |
| 98 | brain vertebrobasilar.mp. |
| 99 | cerebral subarachnoid.mp. |
| 100 | brain subarachnoid.mp. |
| 101 | trauma* brain injur*.mp. |
| 102 | acquired brain injur*.mp. |
| 103 | Brain Injuries/ |
| 104 | brain damage.mp. |
| 105 | Brain Concussion/ |
| 106 | brain hemorrhage.mp. |
| 107 | Brain Injury, Chronic/ |
| 108 | Cerebral Hemorrhage/ |
| 109 | Craniocerebral Trauma/ |
| 110 | Intracranial Hemorrhage, Traumatic/ |
| 111 | Brain Hemorrhage, Traumatic/ |
| 112 | Encephalitis/ |
| 113 | Meningitis, Viral/ |
| 114 | meningitis, viral.mp. |
| 115 | Encephalitis.mp. |
| 116 | Brain Abscess/ |
| 117 | Central Nervous System Infections/ |
| 118 | brain infection*.mp. |
| 119 | cerebral infection$.mp. |
| 120 | brain abscess.mp. |
| 121 | Multiple Sclerosis/ |
| 122 | cerebral ischemia.mp. |
| 123 | Parkinson Disease/ |
| 124 | Dementia/ |
| 125 | Epilepsy/ |
| 126 | Neurodegenerative Diseases/ |
| 127 | Brain Diseases/ |
| 128 | Alzheimer Disease/ |
| 129 | Motor Neuron Disease/ |
| 130 | Neuromuscular Diseases/ |
| 131 | Cognitive Dysfunction/ |
| 132 | "Acceptance and Commitment Therapy"/ |
| 133 | "Acceptance and Commitment Therapy".mp. |
| 134 | 132 or 133 |
| 135 | brain injury.mp. |
| 136 | motor neuron* disease.mp. |
| 137 | dementia.mp. |
| 138 | alzheimer.mp. |
| 139 | multiple sclerosis.mp. |
| 140 | parkinson* disease.mp. |
| 141 | epilepsy.mp. |
| 142 | Cognition Disorders/ |
| 143 | cognition disorders.mp. |
| 144 | head injury.mp. |
| 145 | Hematoma/ |
| 146 | hematoma.mp. |
| 147 | Cerebrovascular Disease.mp. |
| 148 | Hypoxia, Brain/ |
| 149 | hypoxia.mp. |
| 150 | Hypoxia-Ischemia, Brain/ |
| 151 | anoxia.mp. |
| 152 | Subarachnoid Hemorrhage/ |
| 153 | neuromuscular disease*.mp. |
| 154 | brain infarction.mp. |
| 155 | brain concussion.mp. |
| 156 | brain injur*.mp. |
| 157 | 80 or 81 or 82 or 83 or 84 or 85 or 86 or 87 or 88 or 89 or 90 or 91 or 92 or 93 or 94 or 95 or 96 or 97 or 98 or 99 or 100 or 101 or 102 or 103 or 104 or 105 or 106 or 107 or 108 or 109 or 110 or 111 or 112 or 113 or 114 or 115 or 116 or 117 or 118 or 119 or 120 or 121 or 122 or 123 or 124 or 125 or 126 or 127 or 128 or 129 or 130 or 131 or 135 or 136 or 137 or 138 or 139 or 140 or 141 or 142 or 143 or 144 or 145 or 146 or 147 or 148 or 149 or 150 or 151 or 152 or 153 or 154 or 155 or 156 |
| 158 | 134 and 157 |
| 159 | Brain Neoplasms/ |
| 160 | brain neoplasm.mp. |
| 161 | 80 or 81 or 82 or 83 or 84 or 85 or 86 or 87 or 88 or 89 or 90 or 91 or 92 or 93 or 94 or 95 or 96 or 97 or 98 or 99 or 100 or 101 or 102 or 103 or 104 or 105 or 106 or 107 or 108 or 109 or 110 or 111 or 112 or 113 or 114 or 115 or 116 or 117 or 118 or 119 or 120 or 121 or 122 or 123 or 124 or 125 or 126 or 127 or 128 or 129 or 130 or 131 or 135 or 136 or 137 or 138 or 139 or 140 or 141 or 142 or 143 or 144 or 145 or 146 or 147 or 148 or 149 or 150 or 151 or 152 or 153 or 154 or 155 or 156 or 159 or 160 |
| 162 | 134 and 161 |
