# Supplementary material for: A scoping review to identify process and outcome measures used in acceptance and commitment therapy research, with adults with acquired neurological conditions
Source: Clin Rehabil. 2022 Dec 20;37(6):808–35. doi: 10.1177/02692155221144554 (PMC10134096; doi:10.1177/02692155221144554)
Supplement: sj-pdf-2-cre-10.1177_02692155221144554 - Supplemental material for A scoping review to identify process and outcome measures used in acceptance and commitment therapy research, with adults with acquired neurological conditions [file sj-pdf-2-cre-10.1177_02692155221144554.pdf]

Appendix 2: Data extraction tool blank example

**Study details**	Study ID	
	Author/s	
	Title	
	Year	
	Date form completed	
	Country	
	Study design	
	Sample size	
	Acquired, neurological condition	
	Overall aim/target	
**Measurement tool N**	Name of measure N	
	Specified as primary outcome, outcome or process N	
**Process/Outcome N**	What is tool measuring? N	
**Time points N**	Measured immediately post intervention? N	
	Number of maintenance time points N	
	Additional timepoints info N	
